# Faith in a Warming World: Religiosity and Climate Change Anxiety in Epilepsy

**DOI:** 10.1002/brb3.71691

**Published:** 2026-08-20

**Authors:** Gülden Atan

**Affiliations:** ^1^ School of Health Van Yüzüncü Yıl University Van Turkey

**Keywords:** climate change anxiety, epilepsy, nursing, psychological resilience, religiousity

## Abstract

**Purpose:**

This study was aimed to assess the relationship between climate change anxiety and religiousness in individuals with epilepsy.

**Method:**

This study was conducted between July and October 2025 with 330 patients at a university hospital in eastern Turkey. The personal ınformation form, the climate change anxiety scale and the ındividual religion ınventory were used for data collection.

**Findings:**

The mean total score on the climate change anxiety scale for individuals with epilepsy was determined to be 16.25 ± 7.88; and the mean total score on the ındividual religiousness scale was 25.791 ± 5.01. A negative and statistically significant relationship was found between the patients' climate change anxiety and individual religiousness scores (*r* = –0.261, *p* < 0.05). It was determined that the level of individual religiousness reduced the level of climate change anxiety (*ß* = –0.261).

**Conclusion:**

In this study, it was determined that the individual religiousness levels of patients with epilepsy were high, while their climate change anxiety levels were low. Furthermore, it was established that as the level of individual religiousness increased, the level of climate change anxiety decreased.

## Graphical abstract

This study examined the relationship between climate change anxiety and individual religiosity among individuals with epilepsy.

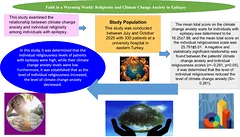

## 1 Introduction

Climate change refers to long‐term alterations in weather patterns and atmospheric conditions resulting from natural processes and human activities. These changes affect environmental variables such as temperature, precipitation, humidity, and wind patterns and have become one of the most significant global public health challenges of the twenty‐first century (Ofremu et al. 2025). Beyond its environmental consequences, climate change has substantial impacts on physical and mental health. It has been suggested that climate change may disproportionately affect individuals with neurological disorders due to impaired thermoregulation and dysregulation of cognitive, sensorimotor, memory, and autonomic functions (Doan and Dhawan 2024). Furthermore, climate‐related factors such as malnutrition, extreme weather events, and changes in atmospheric conditions have been associated with adverse neurological outcomes. For example, fluctuations in temperature have been linked to increased migraine reports in the general population (Ruszkiewicz et al. 2019).

Epilepsy is a common neurological disorder characterized by recurrent seizures and accompanied by neuropsychiatric, cognitive, and psychosocial challenges (Klein et al. 2024). Individuals with epilepsy may be particularly vulnerable to the effects of climate change. Previous studies have shown that environmental factors associated with climate change, including elevated temperatures, increased humidity, and changes in atmospheric pressure, may influence seizure occurrence and severity. Several mechanisms have been proposed to explain this relationship, including genetic susceptibility, alterations in ion channel permeability, activation of proinflammatory pathways, and hyperventilation‐induced alkalosis. In addition, climate‐related conditions may affect the storage, stability, and effectiveness of antiepileptic medications through excessive heat, humidity, or increased sweating (Nunes and da Cunha 2025; Gulcebi et al. 2025).

Awareness of these potential health‐related consequences may contribute to concerns about the future among individuals living with epilepsy. Climate change anxiety is defined as the distress, worry, or fear associated with current or anticipated consequences of climate change. Although climate change anxiety can occur in the general population, individuals with chronic diseases may experience heightened concerns because climate change may be perceived as an additional threat to their health and well‐being (Gago et al. 2024). Previous research has shown that climate change is associated with feelings of anxiety, fear, sadness, uncertainty, and hopelessness (von Gal et al. 2024). Therefore, individuals with epilepsy may experience climate change anxiety not only because of general environmental concerns but also because of the potential impact of climate‐related changes on seizure control, treatment management, and overall quality of life.

Religion and spirituality are important coping resources that help individuals make sense of challenging life experiences and manage emotional distress (Korkmaz et al. 2023). Studies conducted among individuals with chronic illnesses have shown that religious coping is frequently used to manage stress and negative emotions. Similarly, evidence suggests that many patients turn to religious beliefs and practices when facing health‐related concerns and uncertainty (Özcan and Çiftçi 2024; de Medeiros et al. 2024). Therefore, religiosity may also play a role in shaping emotional responses to climate‐related concerns and anxiety.

Studies examining the effects of climate‐related environmental changes on individuals with epilepsy and studies investigating religiosity among individuals with epilepsy are available in the literature (Gulcebi et al. 2025; Özcan and Çiftçi 2024; Wardrope and Reuber 2023; McNicholas et al. 2024; Król et al. 2024; Tedrus 2025). In addition, several studies conducted in different countries have explored the relationship between climate change anxiety and religiosity in general populations (Kwon et al. 2019; Ergun and Rivas 2019; Agusalim and Karim 2024). However, to the best of our knowledge, no study has specifically examined the relationship between climate change anxiety and religiosity among individuals with epilepsy. Given that people with epilepsy may be particularly vulnerable to climate‐related health concerns and uncertainty regarding disease management, understanding the factors associated with climate change anxiety in this population is important and may contribute to identifying psychosocial vulnerabilities, improving supportive care, and informing future healthcare planning for this population. Therefore, this study aims to address this gap by examining the relationship between climate change anxiety and religiosity in individuals with epilepsy.

### 1.1 Study Questions

- What are the levels of climate change anxiety and religiosity among individuals with epilepsy?
- Is there a relationship between climate change anxiety and religiosity among individuals with epilepsy?

## 2 Methods

### 2.1 Study Design, Population, and Sample

This is a cross‐sectional study. It was conducted between July and October 2025 at a state university in eastern Turkey. The population of the study consisted of 544 epilepsy patients who presented to the outpatient clinic at the institution during the data collection period. The required sample size for the population with a known size was calculated using the formula below, with a 95% confidence interval and a ±5% margin of sampling error:

$$n=\frac{Nt^{2}pq}{d^{2}\left(N-1\right)+t^{2}pq},$$

*n* = number of individuals to be sampled; *N* = total population size: 544; *p* = the probability of occurrence of the event being studied = 0.50; *q* = the probability of non‐occurrence of the event (1 – *p*) = 0.50; *t* = the *t*‐value corresponding to the specified degrees of freedom and the chosen margin of error = 1.96; *d* = the desired margin of deviation based on the frequency of the event = 0.05.

$$n=\frac{544.{\left(1.96\right)}^{2}.0.50.0.50}{d{\left(0.05\right)}^{2}\left(544-1\right)+t{\left(1.96\right)}^{2}0.50.0.50}=226.$$

Applying this formula, a minimum of 226 participants was required. Data were ultimately collected from 330 participants, exceeding the minimum requirement. This represents 60% of the population. To achieve the determined sample size, patients were selected using a simple random sampling method based on the hospital list. A post hoc power analysis for the observed correlation between religiosity and climate change anxiety (*r* = –0.261, α = 0.05, two‐tailed) indicated a statistical power greater than 0.99. This demonstrates that the study was sufficiently powered to detect a small‐to‐moderate effect, minimizing the risk of Type II error.

Inclusion criteria were: (1) being 18 years of age or older; (2) ability to communicate verbally; (3) having a diagnosis of epilepsy for at least six months; and (4) willingness to participate in the study.

Exclusion criteria were: (1) being under 18 years of age; (2) unwillingness to participate in the study; and (3) inability to communicate verbally.

### 2.2 Data Collection

Personal Information Form: It was prepared by the researcher based on the data from the literature and contains a total of ten questions regarding patients' age, gender, educational status, marital status, social security status, occupation, and duration of illness, among others (Wardrope and Reuber 2023; Król et al. 2024; Tedrus 2025).

Climate Change Anxiety Scale: It was developed by Stewart in 2021 (Stewart 2021). The scale, whose Turkish reliability and validity study was conducted by Gezer and İlhan, consists of a total of ten items on a 5‐point Likert type. The total score obtained from the scale ranges from 10 to 50, and a higher score indicates a higher level of climate change anxiety. The Cronbach's *α* coefficient was determined to be 0.91 in the Turkish validity and reliability study (Gezer and Ilhan 2021). In the current study, the scale's Cronbach's *α* value was determined to be 0.89.

Individual Religion Inventory: This scale was developed by Zagumny et al. (2012). The Turkish reliability and validity studies were conducted by Ayten in 2013. This single‐factor, 6‐item scale aims to measure the level of influence of religion in individuals' lives, their religious knowledge levels, and the importance they attach to religion. The lowest possible score on the scale is 6, and the highest is 30. The Cronbach's alpha value was found to be 0.85 in the Turkish validity and reliability study (Ayten 2013). In the current study, the scale's Cronbach's *α* value was determined to be 0.90.

### 2.3 Data Analyses

Data analysis for the study was performed using the SPSS 27.0 statistical software package. Descriptive statistics, including frequency, percentage, mean, and standard deviation, were used. Kurtosis and Skewness coefficients were utilized to examine the normality distribution of the data. The *t*‐test, one‐way analysis of variance (ANOVA), and post hoc tests (Tukey, LSD) were also employed. Relationships between the dimensions determined by the scales were examined using Pearson correlation and linear regression analysis. A %95 confidence interval was the basis for all analyses, and the level of significance was set at *p* < 0.05.

### 2.4 Ethical Approval

Approval was granted by the Ethics Committee of a state university in eastern Turkey (Approval No: 2024/14‐11). Furthermore, individuals included in the study were provided with the necessary information regarding the purpose of the study and data collection procedures, and their written informed consent was obtained via an informed consent form. This study was conducted in line with the principles of the Declaration of Helsinki.

## 3 Results

### 3.1 Sample Characteristics

The demographic and clinical characteristics of the participants are presented in Table 1. Of the participants, 32.4% were aged 18–34 years, 56.1% were female, 34.5% were literate, 65.5% were married, and 44.5% lived in urban areas. Nearly half of the participants reported a good income level (49.7%). Regarding clinical characteristics, 34.2% had been diagnosed with epilepsy for 11–15 years, 50.6% had no comorbid disease, and 69.4% were using more than one antiepileptic medication. The mean age was 38.78 ± 11.75 years, and the mean disease duration was 11.98 ± 5.29 years.

**TABLE 1:** Comparison of climate change anxiety and individual religiousness scores of epilepsy patients with demographic characteristics (*n* = 330).

			Climate change anxiety	Individual religiousness
Characteristics	Variables	*n* (%)	*X* ± SD	*X* ± SD
**Age**	18–34	107(32.4)	18.36 ± 8.77	23.09 ± 5.81
	35–44	73(22.1)	16.97 ± 8.42	25.34 ± 4.53
*X* ± SD (38.78 ± 11.75)	45–54	81(24.5)	14.86 ± 7.01	27.11 ± 4.04
	55 and over	69(20.9)	13.86 ± 5.69	28.90 ± 2.16
	Test, *p*		*F*: 5.962	*F*: 26.041
			***p*: 0.001**	***p*: 0.000**
	PostHoc		1 > 3, 1 > 4, 2 > 4 (*p* < 0.05)	2 > 1, 3 > 1, 4 > 1, 3 > 2, 4 > 2, 4 > 3 (*p* < 0.05)
**Gender**	Female	185(56.1)	16.27 ± 8.09	25.62 ± 4.81
	Male	145(43.9)	16.23 ± 7.64	26.01 ± 5.27
	Test, *p*		*t*: 0.049	*t*: –0.692
			*p*: 0.961	*p*: 0.490
**Educational status**	Literate	114(34.5)	14.25 ± 6.63	28.01 ± 2.70
	Primary school	78(23.6)	15.63 ± 7.11	26.35 ± 4.83
	High school	91(27.6)	16.48 ± 7.77	23.88 ± 5.96
	University and higher	47(14.2)	21.70 ± 9.62	23.19 ± 5.17
	Test, *p*		*F*: 11.067	*F*: 19.048
			***p*: 0.000**	***p*: 0.000**
	PostHoc		3 > 1, 4 > 1, 4 > 2, 4 > 3 (*p* < 0.05)	1 > 2, 1 > 3, 2 > 3, 1 > 4, 2 > 4 (*p* < 0.05)
**Marital status**	Married	216(65.5)	14.76 ± 6.93	27.31 ± 3.82
	Single	114(34.5)	19.07 ± 8.80	22.90 ± 5.72
	Test, *p*		*t*: –4.879	*t*: 8.351
			***p*: 0.000**	***p*: 0.000**
**Residential status**	City	147(44.5)	18.30 ± 9.30	25.51 ± 5.17
	District	137(41.5)	14.39 ± 6.28	25.93 ± 5.06
	Village	46(13.9)	15.26 ± 5.43	26.28 ± 4.39
	Test, *p*		*F*: 9.634	*F*: 0.500
			***p*: 0.000**	*p*: 0.607
	PostHoc		1 > 2, 1 > 3 (*p* < 0.05)	
**Income level**	High	164(49.7)	17.25 ± 8.94	25.37 ± 5.40
	Moderate	132(40.0)	15.64 ± 6.89	25.73 ± 4.92
	Low	34(10.3)	13.79 ± 4.87	28.03 ± 2.25
	Test, *p*		*F*: 3.408	*F*: 4.038
			***p*: 0.034**	***p*: 0.019**
	PostHoc		1 > 3 (*p* < 0.05)	3 > 1, 3 > 2 (*p* < 0.05)
**Comorbidities**	Yes	163(49.4)	14.52 ± 6.73	27.78 ± 3.51
	No	167(50.6)	17.94 ± 8.56	23.85 ± 5.49
	Test, *p*		*t*: –4.028	*t*: 7.720
			***p*: 0.000**	***p*: 0.000**
	1–5	32(9.7)	17.94 ± 8.36	23.81 ± 6.34
**Duration of epilepsy (years)**	6–10	105(31.8)	17.34 ± 8.78	24.32 ± 5.40
*X* ± SD (11.98 ± 5.29)	11–15	113(34.2)	15.81 ± 7.56	26.43 ± 4.54
	16–20	49(14.8)	16.27 ± 7.65	27.04 ± 4.31
	21 and more	31(9.4)	12.42 ± 3.54	28.48 ± 1.86
	Test, *p*		*F*: 2.853	*F*: 7.494
			***p*: 0.024**	***p*: 0.000**
	PostHoc		1 > 5, 2 > 5, 3> 5, 4 > 5 (*p* < 0.05)	3 > 1, 4 > 1, 5 > 1, 3 > 2, 4 > 2, 5 > 2, 5 > 3 (*p* < 0.05)
**Number of medications used**	One	101(30.6)	17.74 ± 8.73	22.95 ± 5.57
	More than one	229(69.4)	15.59 ± 7.41	27.04 ± 4.19
	Test, *p*		*t*: 2.296	*t*: –7.362
			***p*: 0.033**	***p*: 0.000**

*F*: Anova test; *t*: independent samples *t*‐Test; PostHoc: Tukey, LSD.

### 3.2 Climate Change Anxiety and Religiosity According to Participant Characteristics

Climate change anxiety scores differed significantly according to age, education level, marital status, place of residence, income level, comorbid disease status, duration of epilepsy, and number of medications (all *p* < 0.05). Higher anxiety scores were observed among younger participants, those with higher education and income levels, single individuals, urban residents, and those without comorbid disease.

Religiosity scores also showed significant differences according to age, education level, marital status, income level, comorbid disease status, duration of epilepsy, and medication use (all *p* < 0.05). Higher religiosity scores were observed among older participants, married individuals, those with lower income levels, participants with comorbid disease, longer disease duration, and those using multiple medications.

### 3.3 Descriptive Statistics

The mean total score of the Climate Change Anxiety Scale was 16.25 ± 7.88, and the mean total score of the Individual Religiosity Scale was 25.79 ± 5.01 (Table 2).

**TABLE 2:** Mean scores of climate change anxiety and individual religiousness (*n* = 330).

	*X* ± SD	Min–max
Climate change anxiety total	16.25 ± 7.88	10–41
Individual religiousness total	25.791 ± 5.017	12–30

SD: standard deviation.

### 3.4 Relationship Between Climate Change Anxiety and Religiosity

Correlation analysis revealed a statistically significant negative relationship between religiosity and climate change anxiety (*r* = –0.261, *p* < 0.01) (Table 3).

**TABLE 3:** Correlation analysis between individual religiousness and climate change anxiety scores.

		Individual religiousness
**Climate change anxiety**	*r*	−0.261**
	*p*	0.000

*p < 0.05; **p < 0.01. Bold values indicate statistically significant results (p < 0.05). Pearson correlation analysis.*

### 3.5 Regression Analysis

Regression analysis showed that religiosity was a significant predictor of climate change anxiety (*F* = 23.920, *p* < 0.05). Religiosity explained 6.5% of the variance in climate change anxiety scores (*R* ^2^ = 0.065). A negative association was observed, indicating that higher religiosity was associated with lower climate change anxiety (*β* = –0.261) (Table 4, 5). The results of the regression analysis are presented in Table 5.

**TABLE 4:** The effect of individual religiousness on climate change anxiety.

Dependent variable	Independent variable	Unstandardized coefficients		Standardized coefficients	*t*	*p*	95% Confidence interval	
		*B*	SE	*ß*			Lower	Upper
Climate change anxiety	Constant	26.818	2.201		12.185	0.000	22.489	31.148
	Individual religiousness	−0.410	0.084	−0.261	−4.891	**0.000**	−0.575	−0.245

*Note*: Dependent variable = climate change anxiety total, *R* = –0.261; *R* ^2^ = 0.065; *F* = 23.920; *p* = 0.000; Durbin Watson Value = 1.883.

*F*: simple linear regression analysis; *t*: independent samples *t*‐test.

**TABLE 5:** Multiple linear regression analysis predicting climate change anxiety.

Variable	*B*	SE	*β*	*t*	*p*	95% CI
**Constant**	20.857	5.937	—	3.513	0.001	9.172 to 32.541
Religiosity	−0.227	0.099	−0.146	−2.306	0.022	−0.422 to –0.033
Age	−0.034	0.055	−0.050	−0.608	0.544	−0.143 to 0.075
Gender	−0.354	0.854	−0.022	−0.415	0.679	−2.034 to 1.326
Educational status	0.310	0.447	0.046	0.693	0.489	−0.570 to 1.189
Marital status	1.784	1.123	0.108	1.589	0.113	−0.425 to 3.993
Residential status	−1.687	0.653	−0.151	−2.583	0.010	−2.971 to –0.402
Income level	−0.914	0.695	−0.078	−1.315	0.189	−2.282 to 0.454
Comorbidities	1.733	1.279	0.110	1.356	0.176	−0.783 to 4.250
Duration of epilepsy (years)	−0.067	0.102	−0.045	−0.656	0.512	−0.267 to 0.134
Number of seizures during the past year	−0.225	0.667	−0.020	−0.338	0.736	−1.538 to 1.087
Number of antiepileptic medications	1.821	1.215	0.106	1.500	0.135	−0.569 to 4.212

*Note*: Model summary: *R* = 0.393, *R* ^2^ = 0.154, Adjusted *R* ^2^ = 0.123, *F* (11,297) = 4.927, *p* < 0.001, Durbin–Watson = 1.942.

## 4 Discussion

In this section, the findings regarding the relationship between climate change anxiety and individual religiousness levels in epilepsy patients are discussed in light of the current literature.

### 4.1 Climate Change Anxiety Scores According to Descriptive Characteristics

The overall level of climate change anxiety among the participants was low. While this finding is consistent with previous studies conducted in Turkey and other populations with chronic illnesses, its significance extends beyond a simple comparison with earlier research (Ağırbaş and Sarıçam 2023; Lawrance et al. 2022; Sharma et al. 2024; El‐Sayed et al. 2025). Climate change anxiety represents an emotional response to perceived environmental threats, but it is also shaped by individuals’ health status, perceived vulnerability, future expectations, and coping resources. Therefore, understanding climate anxiety in people with epilepsy requires consideration of both environmental concerns and the unique challenges associated with living with a chronic neurological disorder.

Although anxiety is often viewed negatively, moderate levels of climate‐related anxiety may promote awareness and encourage adaptive behaviors aimed at environmental protection. This issue may be particularly relevant for individuals with epilepsy because climate change has the potential to affect seizure control through multiple pathways, including heat exposure, sleep disruption, reduced access to healthcare services during extreme weather events, and possible effects on medication management. From this perspective, low levels of climate anxiety may reflect limited awareness of the potential implications of climate change for epilepsy management rather than a complete absence of concern. Therefore, increasing climate‐health literacy among epilepsy patients may represent an important public health objective.

The demographic patterns observed in the present study further support the idea that climate anxiety is closely linked to perceptions of future vulnerability. Younger and single participants reported higher levels of climate anxiety, suggesting that concerns about long‐term environmental changes may be particularly salient among individuals who are actively planning their future lives. Climate change introduces uncertainty regarding health, economic stability, family formation, and quality of life, all of which may be especially relevant for younger adults already coping with a chronic illness (Stewart 2021; Basevitz et al. 2008; Del Valle et al. 2020; Clayton and Karazsia 2020; Tokuç et al. 2024). Thus, age‐related differences in climate anxiety may not merely reflect chronological age but also differences in future orientation and perceived uncertainty.

Similarly, higher climate anxiety among participants with higher educational attainment may indicate greater awareness of environmental risks. Individuals with more education may have increased exposure to information regarding climate change and a better understanding of its potential health consequences (Carmichael and Brulle 2018; Čater and Serafimova 2019). Consequently, greater knowledge may simultaneously increase awareness and emotional concern. This interpretation suggests that climate anxiety should not necessarily be viewed solely as a maladaptive response but may also reflect engagement with environmental realities.

Another noteworthy finding was the higher climate anxiety observed among urban residents. Urban environments are often associated with greater exposure to heat waves, air pollution, and other climate‐related stressors (Gulcebi et al. 2025; Clayton and Karazsia 2020; Kovanci 2023; Kurnaz 2023; Brockmeyer and D'Angiulli 2016; Metin et al. 2024; Korkmaz and Gülsoy 2020) For individuals with epilepsy, these environmental conditions may be perceived as particularly relevant because they can indirectly affect seizure control and overall well‐being. Therefore, urban residence may increase climate anxiety not only through greater exposure to environmental risks but also through heightened awareness of their potential health consequences.

Interestingly, participants with longer disease duration, additional comorbidities, and more complex treatment burdens generally reported lower levels of climate anxiety. Rather than indicating reduced awareness of environmental problems, this finding may reflect a process of priority restructuring. Individuals who have lived with chronic illness for many years often focus their emotional and cognitive resources on managing immediate health concerns (Er and Aktaş 2023; Yadav et al. 2022). Consequently, broader and more distant threats such as climate change may become relatively less salient in daily life. This interpretation highlights the importance of considering climate anxiety within the broader context of chronic disease adaptation and illness burden.

### 4.2 Individual Religiousness Scores According to Descriptive Characteristics

The participants demonstrated relatively high levels of individual religiosity. This finding is particularly relevant in the context of epilepsy because religiosity may serve not only as a personal belief system but also as an important coping resource for managing chronic illness (Rigon et al. 2019). Epilepsy is associated with recurrent uncertainty, concerns about seizure recurrence, social stigma, limitations in daily activities, and worries about the future. Within such circumstances, religious beliefs and practices may help individuals maintain hope, find meaning in their experiences, and develop psychological resilience.

The demographic patterns observed in the present study suggest that religiosity may be closely linked to life experience and illness adaptation. Older participants reported higher levels of religiosity, which may reflect increased engagement with existential questions, greater awareness of personal vulnerability, and a stronger search for meaning over the life course (Özcan and Çiftçi 2024; Król et al. 2024). In individuals living with epilepsy, these processes may be further reinforced by prolonged exposure to illness‐related challenges.

The finding that participants with lower educational attainment reported higher religiosity should be interpreted cautiously. Rather than indicating a simple inverse relationship between education and religiosity, the results may reflect differences in coping preferences and social environments (Schwadel 2024). Religious communities often provide social support, belonging, and practical assistance, resources that may be especially valuable for individuals facing chronic health conditions and socioeconomic challenges

Similarly, higher religiosity among married participants may indicate that religious beliefs operate not only at the individual level but also within family and social relationships. Marriage may reinforce participation in shared religious practices and provide additional opportunities for religious coping. In the context of epilepsy, where social support is a critical determinant of well‐being, these interpersonal dimensions of religiosity may be particularly important.

Participants with longer disease duration, comorbid conditions, and more intensive treatment regimens also reported higher religiosity. These findings suggest that religiosity may become increasingly relevant as illness burden increases. Chronic disease often challenges an individual's sense of control and predictability, and religious beliefs may help restore a sense of meaning, hope, and emotional stability (Fry 2024; Tanrıkulu et al. 2025). Therefore, religiosity may function as an adaptive resource that supports psychological adjustment in the face of ongoing health‐related adversity.

Taken together, these findings suggest that religiosity among people with epilepsy should not be viewed merely as a demographic characteristic. Rather, it appears to represent a multidimensional coping resource that may influence how individuals interpret illness experiences, manage uncertainty, and respond to broader life stressors, including environmental threats such as climate change.

### 4.3 Climate Change Anxiety and Religiousness

One of the most important findings of this study was the statistically significant negative relationship between climate change anxiety and individual religiosity among people with epilepsy. This finding is noteworthy because it highlights how a psychosocial resource such as religiosity may shape responses to a global environmental threat in a population already living with a chronic neurological condition.

The relationship between epilepsy, climate change anxiety, and religiosity can be understood through the concept of coping with uncertainty. Individuals with epilepsy frequently experience uncertainty regarding seizure occurrence, disease progression, treatment outcomes, social participation, and future life plans. Climate change introduces an additional layer of uncertainty by creating concerns about environmental instability, extreme weather events, healthcare accessibility, and future health risks. Consequently, epilepsy patients may simultaneously face both illness‐related and climate‐related uncertainty (Sachdeva 2016). In this context, religiosity may function as a psychological framework that helps individuals interpret threatening events, maintain hope, and preserve a sense of meaning and control despite circumstances that are largely beyond personal influence.

Previous studies have shown that religious coping can strengthen resilience, reduce perceived helplessness, and facilitate adaptation to stressful life circumstances. For people with epilepsy, who already face substantial psychological and social burdens, these coping resources may be particularly valuable. Thus, religiosity may serve as a protective factor that moderates the emotional impact of climate‐related concerns (Kwon et al. 2019; Toros 2025; Harris et al. 2002).

This interpretation also helps explain some of the descriptive findings observed in the present study. Participants with longer disease duration and additional comorbidities reported higher religiosity but lower climate anxiety. Rather than viewing these findings as independent results, they may reflect a common adaptation process. Individuals who have lived with chronic illness for many years often develop coping mechanisms that allow them to manage uncertainty and vulnerability more effectively. Religiosity may represent one of these mechanisms, contributing to reduced anxiety regarding broader environmental threats.

At the same time, the relationship between religiosity and climate anxiety is likely to be complex. Some studies suggest that religious worldviews can reduce environmental anxiety by promoting trust, meaning, and acceptance, whereas others indicate that certain religious interpretations may influence perceptions of environmental responsibility and climate risks. Therefore, religiosity should not be viewed solely as an anxiety‐reducing factor but as a multidimensional construct that shapes how individuals understand and respond to climate‐related challenges (Agusalim and Karim 2024; Khan et al. 2025).

The present study contributes to the emerging literature by examining these relationships in people with epilepsy, a population that has received little attention in climate anxiety research. As climate change is expected to create additional challenges for seizure management, healthcare access, and overall well‐being, understanding factors that influence psychological responses to environmental threats becomes increasingly important. Our findings suggest that religiosity may play a meaningful role in how epilepsy patients perceive and cope with climate‐related concerns. Future longitudinal and qualitative studies are needed to clarify the mechanisms underlying this relationship and to determine whether religiosity directly influences climate anxiety or operates through broader psychological processes such as resilience, coping, social support, and meaning‐making.

### 4.4 Limitations

This study has several limitations. First, specific clinical data such as participants' epilepsy type and seizure control status were not collected. This omission limits our ability to fully evaluate the impact of individual religiousness strategies on epilepsy management. Future research should collect such clinical details for a more comprehensive understanding of the findings. Second, the results of this study are limited to a sample of epilepsy patients from a specific geographical region. Findings conducted in a culturally and religiously distinct region like Van may need to be more generalizable to populations in different socio‐cultural contexts. Therefore, it is recommended that future studies be conducted with larger and more diverse samples in different cultural and geographical settings.

### 4.5 Clinical Implications

Although climate anxiety levels were generally low in our sample, the observed association between religiosity and climate anxiety contributes to a broader understanding of psychological coping processes among individuals with epilepsy. These findings suggest that religiosity may represent one of several factors associated with how patients perceive and respond to emerging environmental concerns, particularly environmental changes that may influence seizure occurrence, seizure severity, and treatment effectiveness. Rather than supporting immediate changes in clinical practice, the results highlight the importance of considering psychosocial and cultural factors when investigating psychological well‐being in chronic neurological conditions. Further longitudinal and multi‐center studies are needed to clarify the clinical relevance of these relationships and to determine whether they have implications for patient care and mental health support.

## 5 Conclusion

The study concluded that the climate change anxiety levels of epilepsy patients were low, and their individual religiosity levels were high. Furthermore, it was determined that as the individual religiosity levels of epilepsy patients increased, their climate change anxiety levels decreased. The patients' climate change anxiety and individual religiosity scores showed significant differences based on age, educational status, marital status, income level, presence of another disease, duration of epilepsy, and number of medications used. However, place of residence was found to affect only the level of climate change anxiety.

It was determined that single individuals, city residents, and those with high income generally had higher levels of climate change anxiety. Furthermore, married individuals, those with low income, and those with an additional comorbidity were found to have increased levels of individual religiosity. These findings suggest that climate change anxiety and individual religiosity may be influenced by various sociodemographic and clinical characteristics, reflecting differences in perceived vulnerability, life experiences, and coping processes among individuals with epilepsy.

In line with these findings, comprehensive education and counseling services may be beneficial for individuals with epilepsy by considering sociodemographic and clinical factors associated with climate change anxiety. Supportive approaches that address psychological well‐being, uncertainty management, and coping strategies may contribute to the psychosocial adaptation processes of individuals living with epilepsy. Furthermore, healthcare professionals should consider psychosocial and cultural factors, including individual belief systems and existing coping resources, when evaluating the psychological needs of epilepsy patients.

It is recommended that future studies investigate the complex relationship between individual religiosity and climate change anxiety using larger and more diverse samples. Longitudinal and qualitative research designs may help clarify whether religiosity influences climate change anxiety directly or through broader psychological mechanisms such as resilience, social support, coping, and meaning‐making. Such research may contribute to developing more comprehensive strategies to support the mental health and quality of life of individuals with epilepsy who may be affected by increasing environmental challenges.

## Author Contributions

**Gulden Atan**: conceptualization, methodology, investigation, data curation, formal analysis, validation, visualization, writing – original draft, writing – review & editing, project administration. The author has read and approved the final version of the manuscript and is accountable for all aspects of the work.

## Funding

The author has nothing to report.

## Ethics Statement

Approval was granted by the Ethics Committee of a state university in eastern Turkey (Approval No: 2024/14‐11). Furthermore, individuals included in the study were provided with the necessary information regarding the purpose of the study and data collection procedures, and their written informed consent was obtained via an informed consent form. This study was conducted in line with the principles of the Declaration of Helsinki.

## Conflicts of Interest

There is no conflict of interest.

## AI Usage Declaration

The authors declare that no artificial intelligence (AI) tools were used in the preparation of this manuscript.

## Data Availability

The data that support the findings of this study are available from the corresponding author upon reasonable request.
